# Gonorrhoea in Greenland, incidence and previous preventive measures: a review to improve future strategies

**DOI:** 10.1080/22423982.2017.1350092

**Published:** 2017-07-26

**Authors:** Sine Berntsen, Anders Peder Højer Karlsen, Michael Lynge Pedersen, Gert Mulvad

**Affiliations:** ^a^ Queen Ingrid Health Care Center, Nuuk, Greenland; ^b^ Greenland Center of Health Research, Institute of Nursing and Health Science, University of Greenland, Nuuk, Greenland

**Keywords:** Gonorrhoea, STI, STD, Greenland, Indigenous, peer-to-peer, story telling, sexual health, primary prevention, sexual education

## Abstract

Gonorrhoea continues to be a significant health challenge in Greenland. The aim of this study was to describe the development of gonorrhoea in Greenland through time including incidence rates and previous measures taken to address the challenge. A systematic literature search in PubMed, Embase and The Cochrane Library was conducted. Furthermore, local archives were searched in the Health Clinic in Nuuk for relevant literature. From the 1940s the incidence of gonorrhoea increased steadily with a steep incline around 1970, possibly as a consequence of changes in living conditions and urbanisation. Significant declines in the incidence were seen the late 1970s and again in the late 1980s, most likely in the wake of an outbreak of ulcus molle/chancroid in the 1970s and as a result of focused education in venereology for Greenlandic nurses in the late 1980s combined with the stop-AIDS campaign. Since the early 1990s the incidence of gonorrhoea in Greenland has not risen to previously high levels. However, the incidence remains high and with a gradually increasing trend. Prevention intervention strategies such as peer-to-peer sexual education, storytelling and involvement of parent/guardian in sexual education of the youth could be appropriate approaches to improve sexual health in Greenland.

## Introduction

Gonorrhoea continues to be a significant health concern worldwide, due to the high incidence of infections and the increasing levels of antimicrobial resistance (AMR) [1].

In Greenland, gonorrhoea and other sexually transmitted infections (STIs) have been a major health challenge for several decades [2]. The incidence of gonorrhoea in Greenland has been estimated periodically within the last 60 years [2–5]. However, an updated comprehensive overview of the incidence has not been created.

In 2014, a total of 1,550 new cases of gonorrhoea were reported in Greenland, with an incidence rate of 2,754 per 100,000 persons. In comparison, the incidence rate was 915 per 100,000 persons in 2005, demonstrating a substantial increase over the last decade and underlining the need for continued focus on gonorrhoea in Greenland [3].

In addition, development of *Neisseria gonorrhoeae* (GC) strains resistant to first-line treatment has evolved continuously [6–10], challenging a health care system delivering high-quality primary health care to a population of 56,000 inhabitants, widely geographically spread in 18 towns and approximately 60 settlements, around the coastline of the biggest island in the world.

Different preventive initiatives have been attempted to decrease the incidence of gonorrhoea, including primary prevention strategies, changes in organisation of health care, new diagnostic strategies, changes in medical treatment and regimes of screening and contact tracing.

However, a truly effective approach to prevent and control the spread of gonorrhoea and other STIs in Greenland remains unknown. A valuable step towards developing such a strategy may be to portray an overview of the incidence of gonorrhoea over the years and to explore earlier preventive measures and concerns regarding gonorrhoea.

Thus, the aim of this study is to give a historical overview of gonorrhoea in Greenland, including changes in incidence rates, earlier concerns and attempts to address the challenge.

## Methods

### Literature search

A systematic literature search in PubMed, Embase and The Cochrane Library was conducted in March 2016. For each database we used the search string: “((Nuuk OR Greenland) AND (gonorrhea OR gonorrhoea OR gonorrhoeae OR STD OR STI OR venereological OR venereal OR sexual))”. Gonorrhoea and venereal were also included as MeSH Terms. For each acquired article the reference list was screened for eligible articles. Further, local archives were searched in the Health Clinic in Nuuk for relevant literature.

All studies were screened for relevance on title and abstract and subsequently downloaded through local access to databases, Google Scholar and ResearchGate.

### Inclusion criteria

Inclusion criteria were all study types concerning gonorrhoea and STIs conducted in Greenland. The primary end-points were incidence, sexual health, social and cultural aspects and treatment including AMR patterns of GC.

## Results

The systematic database search provided 197 articles, of which 92 were duplicates. Screening on title and abstract provided 41 articles of some relevance. Some 14 articles were irretrievable; however, all were published before 1993 and the majority were short statuses on incidence. In total, 27 articles were successfully acquired in full text and a further 22 were retrieved through local archives. Ten eligible articles were found from the reference lists of relevant articles, and 36 articles were finally included for the descriptive analysis. The vast majority of included trials were observational, cohort or qualitative studies or expert opinions on gonorrhoea and STIs.

**Figure 1.**
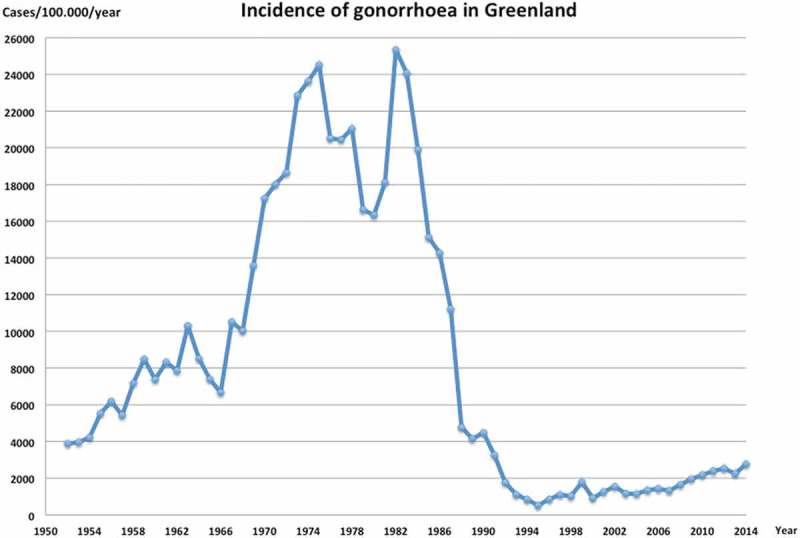
The highest incidence was reported in 1982, but a significant decrease was achieved with campaigns against venereal diseases. Source of annual reported cases of gonorrhoea; 1952–1974: From, E. “Some aspects of venereal disease” [2], 1975–1990: Annual report of gonorrhoea in Greenland [4], 1991–2014: www.bank.stat.gl [3]. Source of population counts; Grønlands statistik [11]

**Figure 2.**
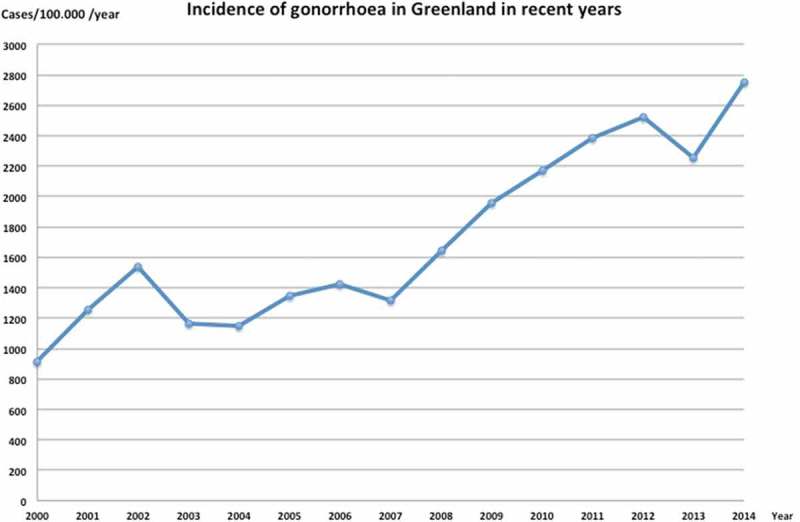
Incidence of gonorrhoea in Greenland from 2000 to 2014. There seems to be a tendency towards continued increase in the incidence of gonorrhoea. Source of annual reported cases of gonorrhoea; Statbank Greenland [3]. Source of population counts; Grønlands statistik [11]

The annual incidences of gonorrhoea from 1951 to 2014 and from 2000 to 2014 are presented in Figures 1 and 2, respectively.

### Incidence

The first cases of gonorrhoea in Greenland were described in 1864 among Greenlandic females in Ivigtut, a former cryolite mine town in southern Greenland. In the early years it was portrayed as a disease found only among European men and the Greenlandic women [12,13]. However, in 1874 the first cases of gonorrhoea in Greenlandic men were described. Over the next 50 years only a few sporadic cases were reported. In 1913–14 the first small epidemic was reported in Greenland; 60 people in the Qaqortoq (Julianehaab) district were treated for gonorrhoea. Most of the infected were married and many were relatively old (above 30 years).

In 1940 Bertelsen described some characteristics of gonorrhoea in Greenland; the female/male ratio was 1:1, while in Denmark it was 1:2.5. Further, 25% of infected female patients were below 15 years of age. In Denmark this number never exceeded 2.5% at the time. Bertelsen stated some obstacles in the fight against gonorrhoea. Low recognition of symptoms and signs of gonorrhoea (especially in women), communication barriers between patients and doctors and high number of sexual partners were some of these obstacles. In addition, contact tracing was difficult, due to the large number of infected married persons (risk of conflicts in a small community) [12].

From the 1940s the prevalence of gonorrhoea in Greenland steadily increased. As demonstrated by Figure 1 there was a steep increase in incidence from 1966 to 1974; around 12,000 cases were reported in 1974. The increase was most pronounced among 16–19-year-olds, especially among females. Gonorrhoea was now a young person’s disease. Furthermore, the male/female ratio changed from 1:1 to 1.5:1 [2,14].

The incidence of re-infections at the time was high; among teenagers diagnosed with gonorrhoea, half would be re-infected within the first year [15].

The increased incidence continued until the late 1970s, where a significant decrease was observed, supposedly due to increased focus on venereal diseases after an outbreak of the painful infection ulcus molle (chancroid) [14]. In 1982 there was a new peak and 13,046 cases were reported – the highest number of cases reported in Greenland to date. In the following years the rate decreased, especially in 1987–1988, most likely due to markedly increased awareness of STIs, education in venereology for Greenlandic nurses and the fear of AIDS. Safe sexual practices among the Greenlandic population were improved, such as increased use of condoms [16]. In 1990 an intervention strategy was introduced involving treatment of all sexual contacts. In 1995 the reported incidence of gonorrhoea was 286 cases – the lowest number reported in years. From then the rate gradually increased again until the year 2000, when there was a decline from 995 cases in 1999 to 515 cases in 2000 [3].

From 2000 the rate has steadily increased up to the current level (Figure 2).

### Treatment and antibiotic susceptibility

Treatment of gonorrhoea has varied considerably over the years, from injection of potassium permanganate into the urethra at the beginning of the 20th century [17] to the current treatment with cephalosporins (a historical overview of treatment and AMR is presented in Table 1). This is mostly due to AMR in GC, which became apparent shortly after the introduction of antimicrobials into clinical practice.

**Table 1.** Historical keypoints in the development of gonorrhoea in Greenland.

Year	Treatment, resistance patterns and social aspects of the disease.
1806	**Social/cultural**: Ship crews were tested for venereal diseases before departure from Denmark, to prevent transmission to the Greenlandic population [13].
1864	**Social/cultural**: The first registered cases of gonorrhoea in Greenland [12].
1933	**Treatment**: A potassium permanganate solution was instilled by the patient himself in the urethra 3–4 times daily for 3 weeks as an emergency treatment until medical attention by a doctor was possible [17].
1962	**Treatment**: I.m. single-dose penilente forte 2.4 mio. IE. If penicillin allergy: tetracycline 250 mg x 4 daily for 5 days. **Resistance**: 26% of patients treated with penicillin were not cured. In most of these patients a prolonged penicillin cure with a higher dose was able to treat the infection [7].
1965	**Treatment**: I.m. benzylpenicillin 5 mio. IE + oral probenecid 1 g single dose was introduced in some areas of Greenland. This was later called “The Greenland method”. Authors suggest that all patients treated for gonorrhoea within the past 6 months and all single persons aged 16–29 years should receive after-treatment on the same day (“G” day) throughout the country to eradicate the disease [18].
1964–78	**Resistance**: Reduced sensitivity to penicillin analysed by strains was reported in 33% from 1964–1970, in 15% from from 1971–1977, and in 33% in 1977–1978. In the period 1971–1977 the standard treatment was the Greenland method. Multiresistant strains were observed in the same period [9,10].
1977	**Treatment**: I.m. cefuroxime 1.5 g or cefuroxime 0.75 g + probenecid 1 g [9].
1977	**Social/cultural**: 57% of teenagers diagnosed with gonorrhoea were re-infected within one year after the primary treatment [2].
1983–85	**Treatment**: Oral benzylpenicillin 5 mio. IE + probenecid 1 g single dose. **Resistance**: Treatment failure due to penicillinase-producing organisms did not seem to occur [8].
1986–95	**Social/cultural**: Stop-AIDS campaign. Due to information about condoms in relation to the stop-AIDS campaign the rate of Greenlandic women aged 20–29 ever to have used a condom increased from 17 to 48%. Greenlandic women had around 4 times more sexual partners compared with Danish women. For 20–39 year old Greenlandic women 53% had had more than 20 sexual partners and 22% reported more than 40 sexual partners. Corresponding Danish rates were 3.6 and 0.3% [18].
1988–93	**Social/cultural**: Specialist in venereology employed in Greenland. Education of Greenlandic nurses around Greenland in venereology.
Mid til late 1990s	**Social/Cultural**: Sex Pilots. Aasiaat. An attempt from the Greenlandic government to provide peer-to-peer-led sexual health education. The programme has not been structurally evaluated [20].
1994	**Treatment**: Oral ciprofloxacin 500 mg single dose [8].
1998–99	**Resistance**: 60% of gonococcal strains were less susceptible or resistent to penicillin. Decreased susceptibility to ciprofloxacin was reported in only 1.5%[8].
2009–12	**Social/cultural**: Inuulluataarneq (Having the Good Life). STI educational intervention with Greenlandic adolescents 15–19 years old and their parents/guardian. Implemented in Paamiut and Uummannaq [21,22].
2012	**Social/cultural**: SexInuk project in Nuuk (and a few classes in Sisimiut, Ilulissat og Tasiilaq.) Peer-to- peer education at the public schools [23].
2013	**Resistance**: During a 35-day period, 32 isolates of *Neisseria gonorrhoeae* strains were collected in Nuuk. All isolates were susceptible to ciprofloxacin, suggesting that resistance was rare [24].
2014	**Resistance**: The first ciprofloxacin-resistant gonorrhoea isolate was found in January 2014 and soon followed by more. In a sample of 85 positive cultures 32% were resistant [6]. **Treatment: I**.m. ceftriaxon 500 mg and oral azithromycin 2 g.

In 1962 Boggild demonstrated that AMR to penicillin was becoming more prevalent; treatment failure was seen in 27% of cases, and 86% of GC strains showed reduced sensitivity [7]. In 1964 Lomholt and Berg also found reduced sensitivity to penicillin in GC strains, leading to the introduction and implementation of “the Greenland method” consisting of benzylpenicillin 5 g and probenecid 1 g as a single oral dose [9,18].

First-line treatment was changed again in 1972 to pivampicillin 1.4 g combined with probenecid 1 g oral dose. In 1983 “the Greenland method” was reinstated for some years [8]. Through the 1970s and 1980s GC resistance to penicillin continued to evolve. As a consequence, standard treatment was changed in 1994 to an oral single-dose administration of ciprofloxacin 500 mg.

In 2002, Dragsted et al. demonstrated less susceptibility or resistance to penicillin in 60% of a group of GC strains isolated in Greenland, and it was confirmed that penicillin should not be used as a first-line drug. Decreased susceptibility to ciprofloxacin was only seen in 1 out of 61 strains [8].

Two recent studies on ciprofloxacin susceptibly in GC in Greenland have been performed; one in 2012 and another in 2014 [6,24]. The first study reported full ciprofloxacin susceptibility among 35 GC isolates. The next study was performed on a larger population and demonstrated a rapid decrease in ciprofloxacin susceptibility among GC isolates in Nuuk within a short period. The percentage of ciprofloxacin-resistant GC strains in the later study was stated as being in same range as reported in other European countries, which led to a change in first-line treatment.

In Greenland the current treatment for gonorrhoea follows the American and European treatment guidelines, which dictates an intramuscular injection of ceftriaxone 500 mg, combined with an oral dose of azithromycin 2 g.

### Diagnosis

Previously the diagnosis of gonorrhoea was based on microscopy and culture of GC from swabs. Since 2011 the diagnosis of gonorrhoea in Greenland has been performed on urine samples with nucleic acid amplification tests (NAATs) by strand displacement amplification (Becton Dickinson ProbeTec). This relatively easily obtained test is offered to all attendees at the Health Care Center and has lead to an increase in number of tests performed, from approximately 17,000 tests in 2010 to over 19,000 tests in 2015.

NAATs provide results faster and are less demanding than culturing the bacteria. Furthermore, the tests are highly sensitive to both symptomatic and asymptomatic infections [25]. In European guidelines, urine samples are the recommended sample for men. In women, urine samples offer a lower sensitivity than swabs from the genital tract and are not the preferred sample; however, they are easier to obtain. The positive predictive value of a test, for example NAAT, is influenced by the prevalence of the disease, for example gonorrhoea, in the tested population. Because the incidence of gonorrhoea in Greenland is high, a high prevalence of gonorrhoea in the population is suspected as well. Therefore NAAT is thought to be a useful test in the Greenlandic population [25].

Culture is the only diagnostic test that enables antimicrobial susceptibility testing. To monitor AMR it is therefore necessary to maintain the capacity for testing by culture. The switch from culture to NAATs in Greenland means that routine AMR testing of all samples is no longer performed.

However, as previously stated, 2 studies on ciprofloxacin susceptibly in Nuuk have been presented since the introduction of NAATs [6,24], and monitoring of ciprofloxacin susceptibility has continued since the first study in 2012 among all men who test positive for gonorrhoea with NAAT in Nuuk.

### Contact tracing

At the Health Care Center in Nuuk it is routine to contact a patient by telephone if he/she has a urine sample that tested positive for gonorrhoea (or chlamydia). The patient is offered a test for hepatitis, HIV and syphilis, and the importance of condom use is discussed with the patient. Furthermore, the patient is encouraged to notify all their sexual contacts within the past 3 months. In Greenland, strategies for contact tracing varies from region to region. In some areas the health care providers take care of contact tracing (provider referral), while in other areas, such as Nuuk, partner notification is used (patient referral).

### Registration

Previously, registration of gonorrhoea cases was done through weekly reports to the chief medical officer from every health clinic in Greenland. Since 2008, the report of gonorrhoea incidence has been based on annual extraction of data from the laboratory system used at The Central Laboratory at Queen Ingrid Hospital, serving all health care centres in Greenland.

### Earlier views on the spread of STIs, sexual behaviour and attempted preventive strategies

Aagaard Olsen was one of the first to investigate sexual health in Greenland in the 1960s. He found that there was a high number of sexual partners and early sexual debut among Inuits. In 1976, when the incidence of gonorrhoea was high, Aagaard Olsen emphasised a need for increased focus in Greenlandic health care politics on preventive measures against STIs. He suggested screening all patients for new infections over a longer period of time following diagnosis and treatment. At the time it was customary to monitor gonorrhoea patients for relapse over a few weeks following treatment. He argued that the treatment was quite effective (which made testing right after treatment unnecessary) and that treated patients often got re-infected in the months following treatment. Local health care personnel around Greenland were to perform screening and diagnostics, which both ethically and economically would be an advantage. According to Aagaard Olsen it would hardly be possible, over the next few years, to change the psychological mechanisms behind the high sexual activity, and thus the aim of a preventive measure should be increased self-observation for signs of disease and regular screening for asymptomatic STIs [26].

In 1980, when the massive increase in gonorrhoea incidence was evolving, specialist in venereology From offered his view on the spread of venereal diseases in Greenland [2]. He suggested that the basic cause was social problems triggered by change to a modern industrial society, and stated that the Greenlandic population had not been given time to keep pace with the developments. He suggested that this might have lead to a higher number of sexual partners, more violence and abuse of alcohol. In addition, urbanisation gave rise to higher concentrations of sexually active individuals.

In 1985 Bardin et al. described a high incidence of HLA-B27 in the Inuit population, leading to increased risk of HLA-B27-related complications of gonorrhoea such as arthritis [27].

Between 1986 and 1988 an information campaign on AIDS was conducted in Greenland. During this period Kjaer et al. found a change in sexual behaviour in the Greenlandic population. The authors reported a significantly increased prevalence of individuals ever having used condoms among Greenlandic women aged 20–29. The incidence of gonorrhoea in Greenland was around 3 times lower in 1988 than in 1986. This decrease in incidence may have been partly due to more frequent condom use [19].

In 1994 Misfeldt reported a systematic intervention strategy against STIs initiated in 1986 in Greenland consisting of (1) information and education, (2) enhancement of the attention of the medical profession to STIs, (3) critical evaluation of methods of examination and treatment, (4) carefully performed partner notification and the use of treatment of contacts, and (5) continued public information about the results of these efforts. Education was established in schools. Instructions for STI examination and treatment were renewed, special training was offered to health care personnel, and in 1988 a venereologist was employed in Greenland. There was an employed venereologist in Greenland until around 1993. The venereologists and their teams, among other things, focused on education of the Greenlandic nurses in the towns and settlements around Greenland. There was a general lack of interest in STIs from the physicians, and the venerology teams found that, when trained Greenlandic nurses were given the opportunity, they were very effective in bringing down the incidence of gonorrhoea by early diagnosis, partner tracing and prompt treatment. This was effective without changing the sexual habits of the population.

Misfeldt reported an almost constant decline in the incidence of gonorrhoea after 1986, but he stated that it was uncertain whether the reduction was due to change in sexual behaviour (for example, increased use of condoms) or improved treatment. He concluded that it was still necessary to influence the general Greenlandic attitude towards sexual behaviour in order to further reduce the incidence of STIs [28].

In 1990 Olsen et al. argued that sexual behaviour was not an isolated part of social and cultural life, and that sexual norms changed when societies changed. He stated that changes in sexual behaviour were only possible if people believed that changes were necessary for maintaining their own health and survival. Thus, in the campaign against AIDS it had been easier to raise public awareness, because AIDS was a sexually transmitted disease with deadly outcome [29].

In a testimony from 1991, Joergensen stated that the most important action in preventing STIs was to influence the attitude towards the general behaviour between partners in a relationship, and not just sexual life [30]. He argued that one of the challenges was the liberal and carefree attitude towards the sexual act, combined with the general reserve in talking about sexual matters. A low sexual debut age and high incidence of rape, as well as jealousy, fear and coercion, were discussed as matters that could all influence the spread of STIs [14].

From the mid 1990s it seems there were some changes in perspective when looking at STI prevention in Greenland. Until then, there had been very little focus on adapting prevention intervention for a Greenlandic context and taking social, cultural and familial matters into account. Focus had been on treatment, diagnosis and tracing, and on attempting to change sexual behaviour – all from a very Western point of view.

From the mid to late 1990s the programme “The Sex Pilots” took place in the Greenlandic town Aasiaat. The programme was established by a Danish nurse, PAARISA (Office of Health and Preventive Measures in Greenland) and the local municipality. The goal was to provide peer-to-peer sexual health education. Unfortunately, however, the programme was supposedly not structurally evaluated afterwards [20,23], and it is difficult to conclude anything from this programme.

In 2015 Homøe et al. evaluated the voluntary project “SexInuk” in relation to peer-to-peer education with a focus on sexual health. The goal was to improve sexual health among young age groups in Greenland by educating Greenlandic students, who then educated pupils in the Greenlandic public school system (7–10 grade) about STIs, anatomy, contraceptives, etc. in the native language. The authors concluded that it was possible to raise awareness and to create a well-functioning team of peer educators by recruiting Greenlandic students who spoke Greenlandic and had knowledge of Greenlandic cultural values. They concluded that further research was needed to investigate how peer-to-peer education could improve sexual and reproductive health in Greenland [23]. The SexInuk programme, where Greenlandic nursing students teach about sexual health in public schools, is still active today.

Most of the known effective preventive strategies towards STIs have been developed in non-Arctic environments that often differ both culturally and socially from Arctic communities, such as the Greenlandic [31,32]. As mentioned earlier, it has been hypothesised that social, cultural and environmental factors need more attention if future preventive strategies are to have significant effect.

The project Inuulluataarneq (Having the Good Life) was implemented from 2009 to 2012 in the Greenlandic cities Paamiut and Uummannaq. It was an STI educational intervention with Greenlandic youth and their parents/guardians. They used community-based participatory research (CBPR) principles to design, implement and evaluate the project, which involved 2 components. The first was STI educational intervention combined with STI testing, tracking and treatment [21]. The second component was parent/guardian education in sexual health communication with adolescents [22].

From research carried out between 2007 and 2009, Gesink et al. reported a lack of communication with parents or guardians about topics related to sex [31,33].

In relation to Inuulluataarneq, Rink et al. stated different matters that may influence sexual behaviour and the spread of STIs in Greenland: little or no direct communication in families about sex, prevalent conflict avoidance, avoidance of awkward situations in general and a cultural norm of fluid and open kinship networks to raise children and youth. Discussions with a parent/guardian about sex, relationships, love, use of condoms and how to avoid getting STIs were reported as being important for the youth in the prevention of STI [21]. Rink et al. found that increasing the youth’s ability to speak with a parent/guardian about sexual health had the potential to reduce sexual risk-taking behaviours and STIs [22].

Intensive short-term education and intervention by a trained community member was suggested.

In Greenlandic public schools, sexual health education is mandatory [23]. However, as Homøe et al. highlighted in their study, the adequacy of this education is unknown. The authors stated that preferably the education should include information about anatomy, contraception and STIs, as well as sensitive topics such as love, sexuality and relationships [23].

## Discussion

Several attempts have been made through the years to bring down the incidence of gonorrhoea and other STIs in Greenland. However, the incidence is still high and gradually increasing.

The current treatment with cephalosporins, a “last-line” treatment option, is threatened as resistant strains of GC have begun to develop internationally [34,35]. The emergence of decreased susceptibility of GC to the cephalosporins, together with AMR already demonstrated to penicillins, sulphonamides, tetracyclines, quinolones and macrolides, makes GC a multidrug-resistant organism [36].

There is generally broad consensus that widespread information about STIs, timely diagnosis/treatment, contact tracing and easy access to health care are factors that can help prevent spread and reduce the number of STIs. These methods have proven successful in Greenland before [2,16,18]. Today, easy and effective diagnosis is available with NAATs.

As presented in Figure 1 and as previously stated, a steady increase in the incidence of gonorrhoea was seen from the 1940s, perhaps due to better and increased testing. From 1966 to 1974 the incidence rose steeply, as a consequence of profound changes in living conditions and urbanisation followed by a huge variety of social problems, as described by From and mentioned earlier in this paper [2,37]. It is a known fact that transmigration to urban areas increases the risk of STIs in Indigenous populations [38].

Two significant decreases in incidence have occurred through time, first after an epidemic of ulcus molle (chancroid) in 1977, and second due to the efforts of venereologists and educated Greenlandic nurses, combined with general increased awareness about safe sexual practices and STIs in relation to AIDS in the late 1980s.

Since the early 1990s the incidence has not risen to previously high levels (Figure 2). Yet, the incidence is still relatively high in Greenland (2,754 per 100,000 persons) compared with Denmark (23 per 100,000 persons) [39].

As mentioned, one of the reasons for the significant decline in gonorrhoea the late 1980s may be the prevention efforts related to HIV/AIDS at the time. We can undoubtedly learn from the intervention strategies and preventive measures used. HIV is generally no longer considered a death sentence, and as a consequence the fear of getting HIV/AIDS is perhaps not as strong a motivator today. However, training and education in STIs and sexual health of local Greenlandic health care personnel in towns and settlements around Greenland, combined with the necessary means for them to perform this job, is still very relevant today.

Historically, the view on STI prevention in Greenland has until recently had a fairly Western perspective. Not much focus has been put on cultural differences of sexual relationships in Indigenous people and Danish/Western people, respectively.

A different strategy than used earlier is most likely needed to bring down the incidence in Greenland today. Focusing on influencing attitudes towards sex, talking about sex, sexual protection, relationships and even love life could be possible approaches.

Rink et al. highlight storytelling as a way of conveying information. In Greenland, talking about sex was originally often done through storytelling or other indirect styles of communication. Thus, by using this method it could perhaps be possible to keep with Greenlandic traditions [21].

The role of the family in STI prevention is another important aspect that should be a bigger part of STI prevention intervention strategies. Rink et al. found that parents/guardians have a vital role to play in educating their youth about sexual health. Traditionally, family played a greater role in educating young people about topics related to sexual and reproductive health [22].

As Homøe et al. suggest, sexual peer-to-peer education is also something that should be investigated further, as it may have potential to influence the sexual health of young Greenlanders. However, peer-to-peer education has mainly been tested in larger towns, and primarily Nuuk. Peer-to-peer education could be a challenge in the smaller settlements. In these settlements it might be better to focus on good communication within families/kinships about sexual health.

FOXY (Fostering Open eXpression among Youth) is a successful ongoing participatory action research project for young women in the Northwestern Territories in Canada at Nunavut and Yukon. Youth are involved with all aspects of the project, from its development to implementation and evaluation. FOXY strives to promote mental and sexual health and healthy relationships using, among other things, traditional beading, theatre, digital storytelling, photography and music [40].

FOXY has some similarities with the SexInuk project. However, FOXY is a much larger project involving more people and possibly with more funding. Future intervention prevention initiatives in Greenland could, however, be inspired by this project, as it concerns sexual health in Indigenous youth and values traditional communication methods.

As Bjerregaard et al. highlight, it should be kept in mind that intervention models developed under very different circumstances cannot be expected to work in Greenland, with small populations scattered over a huge area and still under rapid transition [41].

New intervention studies/methods are to be considered. The earlier relative isolation of the Greenlanders is breaking down somewhat, and television, telephone and access to the Internet are now more widespread. Therefore it could also be speculated whether outreach through social media could encourage improved sexual behaviour in Greenland, for example promoting condom use.

There is very limited research concerning Greenlandic views on sex, sexual health and sexual behaviour [21,22,32]. However, we could learn from studies in other Indigenous populations. As with other Indigenous populations, rapid Westernisation might have caused the Greenlandic population to partly abandon their traditional medical practices and beliefs and adapt to Western practices. Instructions and stories concerning sexual health and reproduction that used to be shared within the families were perhaps lost. As the Internet, telephone and television became more widespread, there was a shift in sources of knowledge. This was in combination with ceased daily contact with grandparents/other elderly relatives, who no longer felt competent to educate the youth in sexual and reproductive health [42].

Greenlandic views on sex and the changes in how Greenlandic families address sexual health and reproduction should be exploited further in future research.

A factor influencing the incidence of gonorrhoea in Greenland might be the relatively low average age of the Greenlandic population (34 years [3]), with many young people of sexually active age.

The increased incidence observed over recent years may partly be explained by centralised electronic registration of gonorrhoea cases since 2008 and increased diagnostic activity since 2010. However, the reoccurrence of syphilis within the last 5 years indicates that STIs are in fact a serious health problem in Greenland [43]. Furthermore, there is perhaps a risk of under-diagnosing gonorrhoea in women, because urine samples for NAATs in women offer a lower sensitivity than swabs [25]. This may increase the risk of transmission, as individuals with a false negative test may not be as likely to use protection with a sexual partner.

Continual tracking of real-time data on gonorrhoea could be a way of immediately registering changes and connecting them to initiatives in the health care system. This would perhaps make it possible to act when an effective measure is found, and describe the measure thoroughly in the literature for future reference. Currently we are only able to acquire incidence data from the year before from the statistics databank, and thus we can only in hindsight see what was effective.

CBPR, as used in, for example, Inuulluataarneq, has been suggested as an appropriate method for research in the Arctic [32]. This approach might help avoid research projects that are carried out by researchers as experts, who describe sexual health and STIs in the Arctic from an outsider point of view, without the involvement of the Indigenous community. With CBPR the experience, cultural aspects and tradition within the Greenlandic population can be combined with the scientific knowledge of the researchers. This might also stimulate the local community to continue health interventions initiated during a research project.

### Strength and limitations

The major strength of this study is that the majority of studies on GC in Greenland have been reviewed in order to understand the different aspects of GC-caused infections in Greenland. However, as not all identified studies were obtained some information may be missing. Screening of the abstracts indicated that the content would contribute little to the retrieved literature. On the other hand, numerous reports only available in paper from local archives, and local knowledge from health care workers with several years of experience in Greenlandic health care, were included in the review. Hence, we consider the present review on GC in Greenland one of the most extensive to date. A limitation, which should be mentioned, is that not all nationally or locally established sexual health programmes and initiatives have been documented over the years. The extent of such measures and their possible benefits remain unknown. Yet, we believe the most significant initiatives are documented and included.

The view on STI prevention in Greenland through time has, as mentioned, until recently generally been from a Western perspective. Not much thought has been given to the problems arising when attempting to implement the same preventive methods as in Denmark or other Western countries without considering Indigenous viewpoints. This is a limitation in our study. The material that has been documented on gonorrhoea in Greenland through time has mainly been from Western researchers, and this might give our historical oversight a more Western perspective.

As mentioned earlier, the registration of gonorrhoea has varied through the years. The methods used to estimate the incidence of gonorrhoea have changed considerably from the 1950s to the present day. It is likely that today’s methods are more accurate, since the incidence is based on annual extraction of data from the laboratory system. This should be taken into consideration when comparing incidences. It can be speculated whether the inclination to track and treat gonorrhoea and other STIs has changed over time, affected by social, political and economic tendencies.

## Conclusion

Gonorrhoea remains at a high rate with an increasing trend. With the growth of AMR, cephalosporins, otherwise reserved for invasive life-threatening diseases, are now being used as first-line treatment against gonorrhoea. This represents a potential health hazard, as the development of antibiotic resistance among other pathogenic bacteria may increase. Measures such as sensitive diagnostic methods and high diagnostic activity are already implemented in the health care system in Greenland. However, truly effective primary preventive health care strategies remain unidentified.

Abrupt decreases in incidence have been observed before, most evident in the 1970s and 1980s. The present incidence is not at the levels observed in those years, but an effort to change the current upward turn is needed. Ongoing structured evaluation of preventive initiatives, using incidence of gonorrhoea or other STIs as outcome, is suggested.

Furthermore, it is recommended to encourage Greenlandic nurses and other local health personnel to be responsible for STI diagnosis, treatment, tracing and information, by educating them in venereology and giving them the necessary time and resources for their work.

Alternative strategies are needed in order to deal with the increase in gonorrhoea incidence today. More focus should be put on the cultural, familial and social context of sexual health and STI prevention in Greenland. Future STI prevention and research could benefit from a more community-based approach/CBPR, and from involving family and youth in sexual health education programmes including a focus on improving communication skills in families. In conclusion, further exploration of recently suggested strategies, such as storytelling, involvement of family and peer-to-peer sexual education, is very relevant in future research to improve preventive strategies against gonorrhoea and other STIs in Greenland.
